# Prediction of Cesarean Section for Intrapartum Fetal Compromise: A Multivariable Model from a Prospective Observational Approach

**DOI:** 10.3390/jpm14060658

**Published:** 2024-06-20

**Authors:** Blanca Novillo-Del Álamo, Alicia Martínez-Varea, Mar Nieto-Tous, Carmen Padilla-Prieto, Fernando Modrego-Pardo, Silvia Bello-Martínez de Velasco, María Victoria García-Florenciano, José Morales-Roselló

**Affiliations:** 1 Department of Obstetrics and Gynecology, La Fe University and Polytechnic Hospital, 46026 Valencia, Spain; novillo_bla@gva.es (B.N.-D.Á.); martinez_alivar@gva.es (A.M.-V.); nieto_martou@gva.es (M.N.-T.); padilla_carpri@gva.es (C.P.-P.); modrego_ferpar@gva.es (F.M.-P.); bello_sil@gva.es (S.B.-M.d.V.); garcia_mariavictoriaflo@gva.es (M.V.G.-F.); 2 Department of Pediatrics, Obstetrics and Gynecology, Faculty of Medicine, University of Valencia, 46010 Valencia, Spain; 3 Department of Medicine, CEU Cardenal Herrera University, 12006 Castellón de la Plana, Spain; 4 Faculty of Health Sciences, Universidad Internacional de Valencia, 46002 Valencia, Spain

**Keywords:** labor, cesarean section, Doppler study, intrapartum fetal compromise

## Abstract

Objective: A cesarean section for intrapartum fetal compromise (IFC) is performed to avoid potential damage to the newborn. It is, therefore, crucial to develop an accurate prediction model that can anticipate, prior to labor, which fetus may be at risk of presenting this condition. Material and Methods: To calculate a prediction model for IFC, the clinical, epidemiological, and ultrasonographic variables of 538 patients admitted to the maternity of La Fe Hospital were studied and evaluated using univariable and multivariable logistic regression analysis, using the area under the curve (AUC) and the Akaike Information Criteria (AIC). Results: In the univariable analysis, CPR MoM was the best single parameter for the prediction of CS for IFC (OR 0.043, *p* < 0.0001; AUC 0.72, *p* < 0.0001). Concerning the multivariable analysis, for the general population, the best prediction model (lower AIC) included the CPR multiples of the median (MoM), the maternal age, height, and parity, the smoking habits, and the type of labor onset (spontaneous or induction) (AUC 0.80, *p* < 0.0001). In contrast, for the pregnancies undergoing labor induction, the best prediction model included the CPR MoM, the maternal height and parity, and the smoking habits (AUC 0.80, *p* < 0.0001). None of the models included estimated fetal weight (EFW). Conclusions: CS for IFC can be moderately predicted prior to labor using maternal characteristics and CPR MoM. A validation study is pending to apply these models in daily clinical practice.

## 1. Introduction

To achieve a vaginal delivery, the concurrence of two circumstances is needed: first, on the maternal side, the uterus must initiate contractions, either spontaneously or induced by oxytocic drugs, and the soft parts must be modified to allow the fetal descent through the birth canal. Second, on the fetal side, the fetus must have sufficient reserves to withstand the physiological stress of labor, as contractions produce repeated transient fetal hypoxia episodes, which reduce the functional fetal reserve [1]. Each fetus has a different metabolic capacity at the onset of labor, and in cases of an imbalance between fetal demands and placental oxygen supply, fetal hypoxia occurs [1]. In this scenario, and to avoid permanent fetal damage, a cesarean section (CS) for intrapartum fetal compromise (IFC) might be needed to minimize the potential consequences of sustained hypoxia.

At labor, this emergency situation is usually diagnosed with an abnormal cardiotocogram, a test with a subjective interpretation and high false positives [2], and/or using a fetal scalp pH, a more objective but inaccurate test, which has recently been called into question by the NICE guidelines [3]. Furthermore, these techniques are performed intrapartum and do not allow us to anticipate the situation earlier. The classical way to predict a potential shortage of fetal metabolic reserves is using the estimated fetal weight (EFW). However, the literature supports that the EFW is not a good determinant of IFC since it simply defines intrauterine growth restriction according to population centiles without considering the genetic growth potential of each fetus.

When the fetus suffers hypoxia, one of its multiple adaptive mechanisms is the centralization of blood flow to the most noble organs, such as the fetal brain [1]. This situation can be evaluated with Doppler ultrasound measuring the cerebro-placental ratio (CPR), the quotient between the middle cerebral artery (MCA) pulsatility index (PI), and the umbilical artery (UA) PI [4]. In cases of abnormal CPR, we may conclude that the fetus is dilating the cerebral vessels and is, therefore, centralizing the blood flow due to hypoxia.

Concerning the predictive ability of CPR for IFC, the literature shows controversial results [5], and it is still unclear which maternal parameters should also be considered to increase its prediction of CS for IFC [5]. Most of the prediction models have been developed to predict CS for failure to progress (FP) [6,7,8,9], and very few to predict CS for IFC [5]. Accordingly, there is insufficient scientific evidence to advise any of them in clinical practice [5].

This work aims to shed some light on the prediction of CS due to IFC prior to labor.

## 2. Material and Methods

This was a prospective observational study that included pregnancies attending the maternity unit of the Hospital Universitario y Politécnico La Fe between March and December 2023.

Inclusion criteria were singleton pregnancies from 32 + 0 to 42 + 0 weeks, with cephalic presentation, that underwent a Doppler ultrasound, either in a previous consult as part of their routine obstetric control or at admission in the obstetric unit, and subsequently presented a spontaneous or induced onset of labor within one day of ultrasound examination. Therefore, all examinations were performed before the onset of contractions, premature rupture of membranes, or any other circumstance leading to labor. The inductions of labor were due to fetal or maternal reasons following the local protocol [10]. No changes in the management of the patient were carried out due to the study.

The exclusion criteria were twin pregnancies, elective CS, stillbirths, patients with any pathology contraindicating vaginal delivery, and delivery later than 24 h post-examination. All the patients that fulfilled the inclusion criteria and did not have an exclusion one were recruited by the professionals collaborating in the study at their admission to the obstetric unit. Despite the observational nature of the study and the management of the patients as per local protocol, informed consent was signed by the patients according to the hospital’s Research Ethics Committee approval (CVS F6SCFZZK:TI7B5L3Z:NNHFUYB9).

The following variables were collected: clinical and epidemiological data including maternal age, pre-pregnancy weight and height, body mass index, number of gestations, parity, last menstrual period, gestational age (GA) at examination (determined according to the crown-rump length in the first trimester), GA at delivery, interval examination to delivery, smoking habits, onset of labor, in case of induction, reason for it, and type of induction (mechanical ripening, use of prostaglandins, or direct oxytocin induction). The ultrasound examinations included EFW, UA PI and MCA PI. EFW was obtained by transabdominal ultrasound measuring the head circumference, biparietal diameter, abdominal circumference, and femur length according to Hadlock’s equation [11]. EFW and birth weight values were converted into centiles using local population centiles adjusted only for fetal gender [12].

The UA and MCA were evaluated using color and pulse Doppler. The MCA Doppler was obtained by the sphenoid wing close to the Willis circle [4], and the UA Doppler was obtained in a free loop of the umbilical cord [13]. The CPR was calculated as the simple ratio between the MCA PI and the UA PI [4]. All Doppler examinations were performed using ultrasound machines with 2–8 MHz convex probes during fetal quiescence, in the absence of fetal tachycardia, and keeping the insonation angle with the examined vessels as small as possible. CPR, MCA PI, and UA PI values were converted into multiples of the median (MoM), dividing each value by the 50th centile (median) at each GA [11,12,13,14]. CPR medians were represented by the following equations:

CPR 50th centile = −3.814786276 + 0.36363249 × GA (in weeks) − 0.005646672 × GA (in weeks)

While the median of the individual Doppler parameters was represented by the following equations:

MCA 50thcentile = −3.266164164 + 0.368135209 × GA (in weeks) − 0.005251488 × GA

UA 50thcentile = 2.2037 − 0.057955 × GA (in weeks) + 0.00053953 × GA

The patients in the study have been managed according to the hospital and national protocol for labor assistance and induction [10]. In case of induction, depending on favorable or unfavorable obstetric conditions, a direct oxytocic induction or cervical ripening (using prostaglandins or mechanically) was performed. In the case of spontaneous onset of labor, the patient usually evolves without medication [10]. The management was not different among the included patients.

Finally, labor outcome data were collected: mode of delivery (spontaneous vaginal delivery, assisted vaginal delivery, CS for FP, CS for IFC), Apgar score at 5 min, arterial cord pH, birth weight (BW), BW centile, fetal sex, and baby destination (maternal ward, neonatal ward, and neonatal intensive care unit). CS for IFC was defined as CS indicated due to an abnormal cardiotocogram or fetal scalp pH < 7.2.

Descriptive statistics were performed to evaluate the variables collected. Continuous variables were presented as the median and interquartile range (IQR), while categorical variables were presented as absolute and relative frequencies. Comparisons were made using Mann-Whitney U tests for continuous data and Fisher’s exact tests for frequency data.

An univariable regression analysis was initially performed to evaluate the parameters and the study outcomes to select plausible determinants. Later, a multivariable logistic regression analysis was performed to assess the diagnostic ability of the variables for the detection of CS for IFC, with estimates, odds ratios (OR), and OR 95% confidence intervals (IC). Afterward, we obtained the Akaike Information Criterion (AIC), and the ROC analysis provided the area under the curve (AUC) and the detection rate ([DR], sensitivity) for a false-positive rate ([FPR], 1-specificity) of 10 and 5%. This analysis was performed on the entire group of studied patients and those undergoing induction of labor.

Statistics were calculated with StatPlus^®^ (AnalystSoft Inc. Apple., Alexandria, VA, USA) for Mac, version 7, and GraphPad Prism^®^ (GraphPad Software, 225 Franklin Street. Fl. 26, Boston, MA 02110, USA) for Mac, version 9. Significance was established at a *p*-value < 0.05.

## 3. Results

Table 1 shows the description of the population included in the study. Moreover, 336 pregnancies were excluded (28 twin pregnancies, 127 elective CS, 2 stillbirths, 167 patients due to a lack of a complete Doppler examination within 24 h of labor, and 12 patients because they did not have informed consent). In summary, the study included 538 pregnancies, of which most were male fetuses (52%), presenting vaginal delivery (77%). Concerning the initiation of labor, 66.3% started with an induction, while 33.6% presented a spontaneous onset. Most patients were examined and delivered between weeks 39 and 40, with a mean examination-delivery interval of 0.5 days. Moreover, the mean BMI was 24.8, the mean number of gestations was 1.7, and the mean parity was 0.67. Concerning delivery, the mean BW was 3216 g, and the mean BW centile was 42. Finally, 8.9% of the fetuses needed CS for IFC, 0% presented a 5-min Apgar score below 7, 2.2% had a low arterial neonatal pH (<7.1), and 0.4% needed admission to the intensive care unit.

Table 1 also shows the differences between pregnancies with vaginal delivery and pregnancies with IFC. Mothers of fetuses without IFC were taller, had higher parity, and were examined later. In addition, their babies were heavier (EFW and BW), presented a higher CPR, were less frequently induced, were delivered at a later GA, and needed less frequent pediatric support.

Table 2 shows the univariable models for predicting CS due to IFC in all the studied pregnancies. CPR MoM (AUC 0.72, *p* < 0.0001) was the most significant parameter. Other significant parameters were maternal height (AUC 0.68, *p* < 0.0001), parity (AUC 0.60, *p* < 0.05), and EFW centile (AUC 0.63, *p* < 0.01).

Table 3 shows the univariable models for predicting CS due to IFC in pregnancies undergoing labor induction (N 357). Again, CPR MoM (AUC 0.72, *p* < 0.0001) was the most significant parameter. Other significant parameters were maternal height (AUC 0.71, *p* < 0.0001) and parity (AUC 0.60, *p* < 0.05). In this case, the EFW centile was not significant.

**Figure 1:**
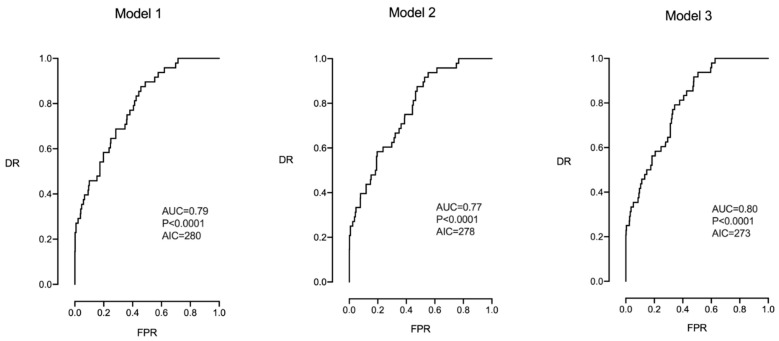
Multivariable analysis in which Models 1, 2, and 3 were evaluated to predict CS for IFC in all the studied pregnancies (N = 538).

Table 4 and Figure 1 show the multivariable logistic regression analysis for predicting CS for IFC in all the studied pregnancies (N = 538). Model 1 included all the studied parameters; Model 2 included only the parameters of Model 1 that were significant; and Model 3 included the parameters that presented borderline significance (smoking and the type of labor onset). The highest prediction and reproducibility (lower AIC) were obtained with model 3, which included maternal age, height, parity, smoking, onset of labor, and CPR MoM (AIC of 273.4, AUC of 0.80, 95% CI (0.74–0.85), *p* < 0.0001, DR 33% for a FPR of 5%, and DR 42% for a FPR of 10%). Of note is that none of the models included the EFW centile.

**Figure 2:**
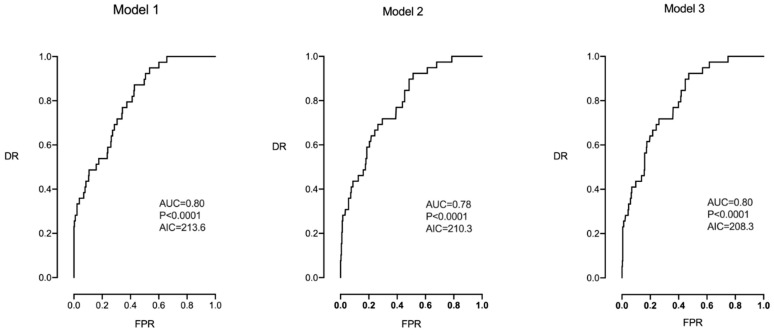
Multivariable analysis in which Models 1, 2, and 3 were evaluated to predict CS for IFC in the group of pregnancies undergoing induction of labor (N = 357).

Table 5 and Figure 2 show the multivariable logistic regression analysis for predicting CS for IFC in the group of pregnancies undergoing induction of labor (N = 357). Model 1 included all the studied parameters; Model 2 included only the parameters of Model 1 that were significant; and Model 3 included the parameters that presented borderline significance (smoking and the type of labor onset). The highest prediction and reproducibility (lower AIC) were obtained with Model 3, which included maternal height, parity, smoking, and CPR MoM, AIC 208.3, AUC 0.80, 95% CI (0.73–0.86), *p* < 0.0001, DR 33% for a FPR of 5%, and DR 44% for a FPR of 10%. Of note is that none of the models included the EFW centile.

## 4. Discussion

This study provides a prediction model of CS for IFC for all gestations and another prediction model for patients undergoing induction of labor. Both include the following variables: maternal height, parity, smoking habits, and CPR MoM. However, the model for the whole population also includes maternal age and the type of onset of labor. Both models achieved a highly accurate prediction (AUC 0.80), although the case of patients undergoing induction presented a higher reproducibility (AIC 208 versus AIC 273), probably because that model included a smaller number of variables.

In the univariate analysis, the variable with the highest prediction ability for both groups was the CPR MoM (AUC 0.72). This agrees with the literature [5], although its prediction ability differs in the different works: some present similar (AUC 0.73) [14], while others present higher (AUC 0.82) [15] or lower accuracies (0.62) [16]. In addition, other authors conclude that CPR is probably unlikely to predict obstetric outcomes due to the presence of other intrapartum determinants [17].

The addition of clinical variables improved the CPR prediction from an AUC of 0.72 to an AUC of 0.80. Of note, and despite its significance in the univariable analysis, no model included the EFW centile since it was not significant in any multivariable analysis—a more robust statistical tool able to control confounding variables, interactions between variables, and reduction of residual error. The literature shows controversial results concerning the importance of the EFW. Some studies indicate that EFW can improve the prediction of CPR [18,19], while others, in line with our work, show no association [20]. In fact, a fetus with growth restriction may present a normal centile (>10th centile), according to local references [15], while it is suffering from intrauterine growth restriction. Consequently, we advocate for a CPR value within normal ranges as a better determinant of fetal well-being according to its dynamism and ability to detect fetal functional reserve.

This study establishes a simple CS for IFC prediction model with an AUC of 0.88 and an AIC of 273.4, resulting, to the best of our knowledge, in the best published predictive model for CS for IFC. Furthermore, in none of the published studies, an extra-specific model is performed for patients undergoing labor induction (AUC 0.80; AIC 208.3). Very few articles in literature have this ambitious goal [5]. The study with the largest sample of patients published up to now for the prediction of emergency CS includes the variables CRP, nulliparity, and induction of labor (as our model), but also the ethnicity and the EFW (AUC 0.77) [21], but did not include maternal age, height, or smoking habits. Another published model (AUC 0.72) for the prediction of operative delivery (not CS) included several variables (gestational age at delivery, parity, CPR, labor induction, EFW, and augmentation using oxytocin), but it included just a selected population (fetuses below the 10th centile for gestational age at 36 weeks of gestation or beyond) [22]. It is also noteworthy that our group published a systematic review of CPR as a predictive factor of CS for IFC, where the predictive value of CPR was very different among the studies due to substantial heterogeneity of the patients and the disparity of the interval examination delivery [5]. In this regard, as the prediction ability of CPR has been recently shown to worsen with this interval [18], we decided to include only patients whose delivery occurred within one day of ultrasound examination.

We recognize some shortcomings in the work: first, the study was carried out in a referral center with frequent obstetric pathology, which could justify the high incidence of CS for IFC (8.9%) [Table 1]. As it is a single-center study, the definition of IFC might differ in other centers, and the model needs internal and external validations before it can be widely applied in clinical practice. Moreover, measurements were calculated by different professionals, and this could influence the results.

## 5. Conclusions

A multivariable model including maternal age, height, parity, smoking habits, CPR MoM, and type of labor onset is able to predict CS due to IFC within one day of delivery. However, in pregnancies undergoing induction, this information can be reduced to only maternal height, parity, smoking habits, and CPR MoM (in both cases: AUC 0.80, *p* < 0.0001). The CPR MoM was the most crucial determinant in this prediction, while the information provided by EFW seemed to be irrelevant. A validation study is pending to apply the models in daily clinical practice.

## Figures and Tables

**Table 1:** Description of the study population (N = 538).

	1-All Pregnancies (N = 538)	2-No IFC (N = 490)	3-Cesarean Section for IFC (N = 48)	2 vs. 3 *
	Mean (SD); Median (1st, 3rd Quartile)	Mean (SD); Median (1st, 3rd Quartile)	Mean (SD); Median (1st, 3rd Quartile)	*p*-Value
Maternal age in years	32.9 (5.6); 33 (29, 37)	32.9 (5.6); 33 (29, 37)	33.5 (5.1); 33.5 (30, 37.7)	NS
Maternal pre-pregnancy weight (kg)	65.6 (13.4); 63 (56, 72)	65.6 (13.3); 63 (56, 72)	66.2 (15.1); 61.5 (56.2, 73.5)	NS
Maternal height (cm)	162.8 (6.3); 163 (158, 167)	163.2 (6.1); 164 (158, 168)	158.7 (6.2); 157.5 (155, 163)	<0.0001
Maternal Body Mass Index, Kg/m^2^	24.8 (5.0); 24.0 (21, 27)	24.6 (4.9); 24 (21, 26.2)	26.3 (6); 24 (22, 30)	NS
Number of gestations	1.7 (1.2); 1 (1, 2)	1.7 (1.3); 1 (1, 2)	1.5 (0.9); 1 (1, 2)	NS
Parity	0.67 (0.9); 0 (0, 1)	0.7 (0.9); 0 (0,1)	0.42 (0.79); 0 (0, 1)	<0.05
Gestational age at examination (week)	39.7 (1.3); 40 (39.3, 40.6)	39.8 (1.1); 40 (39.4, 40.5)	38.9 (2.2), 39.6 (38.3, 40.4)	<0.01
Gestational age at delivery (week)	39.8 (1.3); 40.1 (39.4, 40.6)	39.9 (1.1); 40.1 (39.5, 40.6)	39 (2.2); 39.7 (38.4, 40.4)	<0.01
Interval examination delivery	0.5 (0.5); 0 (0, 1)	0.5 (0.5); 0 (0, 1)	0.4 (0.5); 0 (0, 1)	NS
Estimated fetal weight (gram)	3216 (567.4); 3265 (2893, 3582)	3249 (553); 3295 (2933, 3610)	2879 (610); 2889 (2478, 3363)	<0.0001
Estimated fetal weight centile **	42 (32.7); 37 (11, 70)	43.2 (32.9); 39 (12.7, 72.2)	28.4 (27.7); 26.5 (3, 41.7)	<0.01
CPR	1.65 (0.52); 1.60 (1.28, 1.97)	1.7 (0.5); 1.6 (1.3, 2)	1.3 (0.51); 1.17 (0.94, 1.61)	<0.0001
CPR MoM	0.97 (0.32); 0.94 (0.75, 1.16)	0.99 (0.31); 0.95 (0.77, 1.16)	0.74 (0.29); 0.67 (0.53, 0.94)	<0.0001
Birth weight (gram)	3213 (567.8); 3270 (2898, 3600)	3256 (549); 3300 (2950, 3636)	2775 (576.8); 2785 (2374, 3188)	<0.0001
Birth weight centile **	41.1 (32.7); 38 (11, 70)	43.15 (32.6); 40 (13, 72)	20.2 (26.2), 6 (1, 27.7)	<0.0001
	**N (%)**	**N (%)**	**N (%)**	
Smoking	49 (9.1)	47 (9.6)	2 (4.2)	NS
Male sex	281 (52.2)	254 (51.8)	27 (56.2)	NS
Onset of labor				
Induction of labor	357 (66.3)	318 (64.9)	39 (81.2)	<0.05
Spontaneous onset of labor	181 (33.6)	172 (35.1)	9 (18.7)	<0.05
Apgar < 7 at 5 min	0 (0)	0 (0)	0 (0)	NS
Arterial pH < 7.10	12 (2.2)	8 (1.6)	4 (8.3)	<0.05
Mode of birth				
Cesarean section (failure to progress)	73 (13.5)	73 (14.9)	0 (0)	<0.01
Cesarean section (IFC)	48 (8.9)	0 (0)	48 (100)	<0.0001
Assisted vaginal delivery	111 (20.6)	111 (22.6)	0 (0)	<0.0001
Spontaneous vaginal delivery	306 (56.9)	306 (62.4)	0 (0)	<0.0001
Neonatal destiny				
Maternal ward	503 (93.5)	463 (94.5)	40 (83.3)	<0.01
Neonatal ward	33 (6.1)	26 (5.3)	7 (14.6)	<0.05
Neonatal Intensive care unit (NICU)	2 (0.4)	1 (0.2)	1 (2.1)	NS

Notes: * Mann-Whitney U test, SD: standard deviation, IFC: intrapartum fetal compromise, NS: not statistically significant. ** Estimated fetal weight and birth weight centiles from the references of the Barcelona Clinic hospital.

**Table 2:** Univariable models for the prediction of cesarean section due to intrapartum fetal compromise one day prior to labor (N = 538). CPR MoM was the best parameter.

	Estimate	SE	OR (95% CI)	OR *p*-Value	AUC	AUC *p*-Value
**Maternal age**	0.0197	0.0275	1.0199 (0.9663, 1.0764)	NS	0.53	NS
**Maternal height**	−0.1169	0.0256	0.8896 (0.8460, 0.9355)	<0.0001	0.68	<0.0001
**Maternal weight**	0.0034	0.0110	1.0034 (0.9819, 1.0252)	NS	0.50	NS
**Parity**	−0.4502	0.2247	0.6375 (0.4104, 0.9902)	<0.05	0.60	<0.05
**Fetal sex (male)**	0.1778	0.3047	1.1946 (0.6574, 2.1705)	NS	0.51	NS
**Smoking**	−0.8921	0.7384	0.4099 (0.0964,1.7424)	NS	0.53	NS
**Onset of labor (induction)**	0.8518	0.3817	2.3438 (1.1092, 4.9528)	<0.05	0.53	NS
**EFW centile**	−0.0155	0.0053	0.9846 (0.9745, 0.9949)	<0.01	0.63	<0.01
**CPR MoM**	−3.1470	0.6282	0.0429 (0.0125, 0.1472)	<0.0001	0.72	<0.0001

Notes: SE: standard error, OR: odds ratio, AUC: area under the curve, NS: not statistically significant.

**Table 3:** Univariable models for the prediction of cesarean section due to IFC one day prior to labor in cases undergoing induction of labor (N = 357).

	Estimate	SE	OR (95% CI)	OR *p*-Value	AUC	AUC *p*-Value
**Maternal age**	0.00244	0.0298	1.0024 (0.9455,1.0628)	NS	0.50	NS
**Maternal height**	−0.13225	0.0303	0.8761 (0.8256, 0.9297)	<0.0001	0.71	<0.0001
**Maternal weight**	−0.00808	0.0124	0.9919 (0.9681, 1.0164)	NS	0.54	NS
**Parity**	−0.57145	0.2700	0.5647 (0.3326, 0.9587)	<0.05	0.60	<0.05
**Fetal sex (male)**	0.10383	0.3402	1.1094 (0.5695, 2.1613)	NS	0.51	NS
**Smoking**	−0.85938	0.7472	0.4234 (0.0979, 1.8316)	NS	0.52	NS
**EFW centile**	−0.01050	0.0055	0.9896 (0.9789, 1.0003)	NS	0.59	NS
**CPR MoM**	−2.85858	0.6554	0.0573 (0.0159, 0.2072)	<0.0001	0.72	<0.0001
**Maternal age**	0.00244	0.0298	1.0024 (0.9455,1.0628)	NS	0.50	NS

Notes: SE: standard error, OR: odds ratio, AUC: area under the curve, NS: not statistically significant.

**Table 4:** Multivariable models for the prediction of cesarean section due to intrapartum fetal compromise one day prior to labor (N = 538). Model 3 obtained the best prediction ability.

	Estimate	SE	OR (95% CI)	OR *p*-Value
**Model 1.** All studied parameters.				
Maternal age	0.06169	0.03145	1.06364 (1.00006, 1.13125)	<0.05
Maternal height	−0.13289	0.02976	0.87556 (0.82594, 0.92815)	<0.0001
Maternal weight	0.01648	0.01154	1.01661 (0.99388, 1.03987)	NS
Parity	−0.54588	0.24211	0.57933 (0.36045, 0.93114)	<0.05
Fetal sex (male)	−0.07152	0.33693	0.93098 (0.48099, 1.80193)	NS
Smoking	−1.59112	0.86061	0.20370 (0.03771, 1.10042)	BS
Onset of labor (induction)	0.76041	0.42105	2.13915 (0.93721, 4.88254)	BS
EFW centile	−0.00432	0.00614	0.99569 (0.98378, 1.00775)	NS
CPR MoM	−2.57426	0.66293	0.07621 (0.02078, 0.27946)	<0.001
Intercept	18.19613			
AIC: 280, AUC: 0.79, 95% CI (0.72–0.85), *p* < 0.0001, DR 35% for a FPR of 5%, DR 44% for a FPR of 10%.				
BS: (Borderline significance): *p* = 0.06448 for smoking and *p* = 0.07092 for onset of labor (induction).				
**Model 2.** Significant parameters in model 1				
Maternal age	0.06427	0.03068	1.06638 (1.00415, 1.13248)	<0.05
Maternal height	−0.11838	0.02830	0.88836 (0.84042, 0.93903)	<0.0001
Parity	−0.48508	0.23256	0.61565 (0.39027, 0.97116)	<0.05
CPR MoM	−2.77539	0.63239	0.06233 (0.01805, 0.21526)	<0.0001
Intercept	17.26465			
AIC: 278.3, AUC: 0.77, 95% CI (0.70–0.85), *p* < 0.0001, DR 33% for a FPR of 5%, DR 40% for a FPR of 10%.				
**Model 3.** Significant parameters in model 1 plus smoking and onset of labor (BS in model 1).				
Maternal age	0.05881	0.03088	1.06057 (0.99828, 1.12674)	BS
Maternal height	−0.12789	0.02906	0.87995 (0.83122, 0.93153)	<0.0001
Parity	−0.50124	0.23491	0.60578 (0.38226, 0.96000)	<0.05
Smoking	−1.57965	0.84415	0.20605 (0.03939, 1.07775)	BS
Onset of labor (induction)	0.86853	0.41351	2.38340 (1.05976, 5.36026)	<0.05
CPR MoM	−2.69226	0.62885	0.06773 (0.01975, 0.23230)	<0.0001
Intercept	18.37193			
AIC: 273.4, AUC: 0.80, 95% CI (0.74–0.85), *p* < 0.0001, DR 33% for a FPR of 5%, DR 42% for a FPR of 10%.				
BS: (Borderline significance): Maternal age = 0.05686, Smoking = 0.06130.				

Notes: SE: standard error, OR: odds ratio, AUC: area under the curve, MoM: multiples of the median, CI: confidence interval, AIC: Akaike Information Criterion, DR: discrimination rate, FPR: false positive rate, NS: not statistically significant.

**Table 5:** Multivariable models for the prediction of cesarean section due to intrapartum fetal compromise one day prior to labor in cases undergoing induction of labor (N = 357). Model 3 obtained the best prediction ability.

	Estimate	SE	OR (95% CI)	OR *p*-Value
**Model 1**. All studied parameters.				
Maternal age	0.04569	0.03482	1.0467 (0.9777, 1.1207)	NS
Maternal height	−0.15103	0.03667	0.8598 (0.8002, 0.9239)	<0.0001
Maternal weight	0.00687	0.01289	1.0069 (0.9817, 1.0326)	NS
Parity	−0.71665	0.29288	0.4884 (0.2751, 0.8671)	<0.05
Fetal sex (male)	−0.26751	0.38380	0.7652 (0.3607, 1.6238)	NS
Smoking	−1.51535	0.89371	0.2197 (0.0381, 1.2666)	BS
EFW centile	0.00278	0.00670	1.0028 (0.9898, 1.0160)	NS
CPR MoM	−2.64108	0.72647	0.0713 (0.0172, 0.2961)	<0.001
Intercept	23.03626			
AIC: 213.6 AUC: 0.80, 95% CI (0.73–0.86), *p* < 0.0001, DR 36% for a FPR of 5%, DR 44% for a FPR of 10%.				
BS: (Borderline significance): 0.08997 for smoking.				
**Model 2**. Significant parameters in model 1				
Maternal height	−0.12014	0.03227	0.8868 (0.8324, 0.9447)	<0.001
Parity	−0.62626	0.28615	0.5346 (0.3051, 0.9366)	<0.05
CPR MoM	−2.35199	0.65413	0.0952 (0.0264, 0.3430)	<0.001
Intercept	19.61224			
AIC: 210.3, AUC: 0.78, 95% CI (0.71–0.86), *p* < 0.0001, DR 31% for a FPR of 5%, DR 44% for a FPR of 10%.				
**Model 3**. Significant parameters in model 1 plus smoking and onset of labor (BS in model 1).				
Maternal height	−0.12770	0.03279	0.8801 (0.8253, 0.9385)	<0.001
Parity	−0.64303	0.28911	0.5257 (0.2983, 0.9265)	<0.05
Smoking	−1.48275	0.84867	0.2270 (0.0430, 1.1980)	BS
CPR MoM	−2.49002	0.65890	0.0829 (0.0228, 0.3016)	<0.001
Intercept	21.05541			
AIC: 208.3, AUC: 0.80, 95% CI (0.73–0.86), *p* < 0.0001, DR 33% for a FPR of 5%, DR 44% for a FPR of 10%.				
BS: (Borderline significance): Smoking = 0.08061.				

Notes: SE: standard error, OR: odds ratio, AUC: area under the curve, MoM: multiples of the median, CI: confidence interval, AIC: Akaike Information Criterion, DR: discrimination rate, FPR: false positive rate, NS: not statistically significant.

## Data Availability

Data will be obtained by reaching out to the authors while ensuring the privacy of the patients is maintained.

## Abbreviations

Cesarean section (CS); estimated fetal weight (EFW); birth weight (BW); umbilical artery (UA); middle cerebral artery (MCA); pulsatility index (PI); cerebro-placental ratio (CPR); gestational age (GA); multiples of the median (MoM); odds ratio (OR); confidence interval (IC); area under the curve (AUC); Akaike Information Criterion (AIC); standard error (SE); discrimination rate (DR); false positive rate (FPR).
